# A systematic review and meta-analysis of patients’ exit knowledge and associated factors for drugs dispensed at outpatient pharmacies in Ethiopia

**DOI:** 10.3389/frhs.2025.1436591

**Published:** 2025-02-27

**Authors:** Temesgen Geta Hardido, Christian Kebede, Tamirat Beyene

**Affiliations:** College of Medicine and Health Science, Wolaita Sodo University, Wolaita Sodo, Ethiopia

**Keywords:** Ethiopia, patient, knowledge, dispensed drugs, systematic review and meta-analysis

## Abstract

**Background:**

Patients' knowledge of dispensed drugs is essential to prevent preventable patient mortality and facilitating their recovery from illnesses. Several studies have been conducted in Ethiopia, but the overall level of patients' exit knowledge about the dispensed drugs and associated factors has not been estimated. The objective of this review is to assess overall level patients' exit knowledge of dispensed drugs and associated factors in Ethiopia.

**Methods and materials:**

Only articles published in English were included in this review. PubMed, EMBASE and CINAHL, Web of Science, Google Scholar, Scopus, Ethiopian University Repository Online, and the Cochrane Library are the main databases. The review included cross-sectional studies written in English that met the inclusion criteria. Using a random effects model, the overall level patients’ exit knowledge of dispensed drugs was estimated. Additionally, funnel plots and Eggers' test were used to assess publication bias. STATA version 14 was used to perform all statistical analyzes.

**Results:**

This review included 10 studies involving 3,431 patients in Ethiopia. In Ethiopia, the overall patients' knowledge towards dispensed drugs was 50.73% [95% CI (31.81; 69.66); I2 = 99.4%, *P* < 0.001]. The patients' exit knowledge of dispensed drugs was statistically associated with the education level of the patients and the availability of adequate drug information.

**Conclusions:**

One in two patients has good knowledge of dispended drugs in Ethiopia. Therefore, patients need special attention when dispensing drugs and leaving a health facility. Furthermore, the Ethiopian government, pharmacists, and other stakeholders must take immediate action to improve patients' knowledge about dispensed drugs in Ethiopia and identified factors.

**Systematic Review Registration:**

https://www.crd.york.ac.uk/prospero/display_record.php?ID=CRD42024544256, identifier: CRD42024544256.

## Introduction

According to the World Health Organization (WHO) definition, medicines are rationally used if patients receive medicines that meet their clinical needs, in doses that meet their individual needs, for a reasonable period of time and at the lowest cost to them and their communities (1). However, 67% of the world's population does not have access to essential medicines, more than half of all medications are not prescribed and administered appropriately, and 50% of patients use drugs that have been improperly dispensed (2).

Dispensing is one of the main professional roles of pharmacists in Ethiopia. This is a process of pharmacist-patient interaction that delivers appropriate instructions for the patient about the treatment and prevention of diseases (3). It plays a key role in maintaining the rational use of drugs. Consequently, the outcome of the treatment of patients depends mainly on their adhesion to the medications they are dispensed and lifestyle changes. Pharmacists are responsible for counseling patients after doctor prescriptions. Therefore, adequate counseling provided by pharmacists is essential to maintain a safe and cost-effective use of drugs (3–7). Patients should receive sufficient information and advice on the dispensed drugs, such as the name of the drug, the mode of administration, frequency, benefit, duration, possible side effects, measures to take if the dose is not taken, and other additional precautions (8–11). The lack of essential drugs and inappropriate dosage regimens can cause serious health problems, especially in children and chronic diseases such as cardiovascular disease, diabetes, and epilepsy (12, 13).

Drug errors resulting from inappropriate drug use increase unnecessary costs for patients, caregivers, and health facilities (6). Furthermore, in low-income countries, problems related to drug use include multi-drug use, inappropriate duration of drug, a high or low dose of drugs, and reduce hospital visits due to inaccessible medicines and loss of confidence. The most common reasons for drug misuse are lack of knowledge, lack of adequate information, the burden on health workers, negligence, inadequate staff training, inaccurate diagnosis, and poor patient-dispenser relationship (14–18).

To understand properly why and how to take drugs, for what purpose they take, and for how long time they take, adequate counseling time is required for patients, both by doctors and dispensing pharmacists. In addition to the role of the provider or manufacturer, the patient's level of education and prescription experience can affect their knowledge of the drugs provided. The quality of interactions between the pharmacist and the patient also affects the patient's knowledge of drug usage and their satisfaction level. To ensure that patients know the dosage regimen correctly and thus increase compliance with the correct regimen, it is crucial that an appropriate amount of time is spent in the consultation. Therefore, it is necessary to improve the knowledge of patients about the drugs they use (19–21).

Patient knowledge of drugs is the extent to which they will have knowledge of the drug they will take, including the name of the drug, indication, dose, dosage protocol, side effects, and precautions during treatment, contraindications, interaction between drugs and foods or advice on storage conditions (22). Poor knowledge of prescribed drugs and low compliance with drugs may lead to burdens on public health care. Chronic diseases such as high blood pressure, diabetes, and asthma require cooperation between health care providers and patients to achieve long-term goals of treatment (23).

Inadequate knowledge of the patient about the drug he or she uses may lead to incorrect use, noncompliance with a drug regimen, a reduction in its effectiveness of therapy, and unexpected economic consequences that affect the success of the health system. Lack of information on the drugs dispensed can also lead to unintended overdose and no adherence (24, 25). According to the WHO, more than 50% of all medications in the world are misdiagnosed and 50% of patients cannot use them appropriately (26). Studies conducted among patients in Sri Lanka, Ghana, and eastern Ethiopia showed that they were unaware of the drugs being administered. Factors such as higher education levels, private employee, severe perception of illness, three or more visits, and politeness of the pharmacist, and dispenser guidance were positively associated with the patient's knowledge of the drugs dispensed whereas adequately counseled before discharge, patients' occupation type, and long wait time were negatively associated with the patient's knowledge of the drugs dispensed (22, 25–30).

Additionally, there is a gap in maintaining adequate knowledge of patients about the dispensed drugs. Dispensing is also considered the “exit gate” and the last point of contact of patients with hospital physicians. Unless patients are adequately advised about their treatment and medications at this basic stage, all investigations, resources, and time invested for the patient will be futile. This, in turn, would undermine the health and economic outcomes for both patients and institutions. Therefore, it is essential to continuously assess the patient's exit knowledge of drugs they received (27).

This is the first systematic review of important gaps in knowledge of drugs dispensed among patients in Ethiopia. Although separate studies have been conducted to assess patients' exit knowledge toward dispensed drug in Ethiopia, there are no national data supporting the overall level in Ethiopian outpatients (23, 27, 30–36). Therefore, the objective of this systematic review and meta-analysis is to evaluate the overall level of knowledge of drugs dispensed to patients in Ethiopia and related factors. The results of this study provide general information to reduce morbidity and mortality in patients, improve policy, design strategies, and improve pharmaceutical services. This plays an important role in reducing the morbidity and mortality of patients. The objective of the review was to answer the following research questions. (1) What is the level of knowledge about drugs dispensed and related factors among outpatients in Ethiopia? (2) What are the associated factors that affect the knowledge of the drugs dispensed among outpatients in Ethiopia?

## Methods and materials

A systematic review and meta-analysis was conducted to estimate the overall level of patients' exit knowledge and related factors of drugs dispensed at outpatient pharmacies in Ethiopia.

### Search strategy

Scientific Databases: PubMed, EMBASE and CINAHL, Web of Science, Scopus, and Cochrane Library and Grey Literature: Ethiopian University Repository and Google Scholar were used to search for studies till April 30, 2024. Furthermore, missing data were processed by contacting the appropriate authors. We checked the database at (http://www.library.ucsf.edu) and the Cochrane Library to ensure that this study was not conducted and to avoid duplication of efforts. PROSPERO also registered this review with the registration number CRD42024544256. After confirming that a similar study had not previously been conducted in Ethiopia, a comprehensive search strategy was developed using multiple Boolean operators on standard population exposure and outcome (PEO) questions. The words “or” and “and” were used to combine search terms. The terms “knowledge”, “awareness”, “dispensed drugs”, “dispensed medication” AND “associated factors” or “influencing factors”, “client” OR “patient” OR “outpatient” AND Ethiopia are searched using Boolean operators (Table 1). These papers met the criteria for inclusion in terms of titles, and the abstracts were read in full. Three authors (TG, CK, and TB) carried out the search strategy. All articles retrieved from the database were checked for titles and abstracts before being exported to the EndNote library. These articles met the inclusion criteria in terms of titles and abstracts that were read in full. Three authors (TG, CK, and TB) performed a search strategy. We strictly followed the Preferred Reporting Items for Systematic Review and Meta-Analysis (PRISMA) protocol for this review.

**Table 1:** Example of searches for the scientific databases and grey literatures to assess patients’ exit knowledge and associated factors for drugs dispensed at outpatient pharmacies in Ethiopia.

Databases	Searching terms	Date & time search was done	Number of studies identified
PubMed	(“knowledge” OR “Awareness”, AND “dispensed drugs” [All Fields] OR “dispensed medication” [All Fields] AND “Outpatient” “[All Fields] OR “client” [All Fields] AND “determinants” OR “associated factors” OR “influencing factors” AND [“Ethiopia” (MeSH Terms)]	April 1–30, 2024 at 8:00–11:00 AM	396
EMBASE and CINAHL	(*knowledge* OR *Awareness*) [ti,ab,kw] AND (*determinants* OR *associated* OR influencing factors) [ti,ab,kw] AND (dispensed medication or dispensed drugs) AND (outpatient pharmacies [ti,ab,kw] AND (“Ethiopia” [ ti,ab,kw]	April 1–30, 2024 at 8:00–11:00 AM	72
Scopus	(Title-ABS-KEY) (*knowledge*, OR *awareness*) AND (*dispensed drugs* OR (*dispensed medication*) AND (*associated factors* or *influencing factors*) AND (*client* OR *patient* OR *outpatient*) AND (*Ethiopia*)	April 1–30, 2024 at 8:00–11:00 AM	47
Web of Science	(Title-ABS-KEY) (*knowledge*, OR *awareness*) AND (*dispensed drugs* OR (*dispensed medication*) AND (*associated factors* or *influencing factors*) AND (*client* OR *patient* OR *outpatient*) AND (*Ethiopia*)	April 1–30, 2024 at 8:00–11:00 AM	68
Records identified through other source	Grey literature (Ethiopian University Repository and Google Scholar)	April 1–30, 2024 at 8:00–11:00 AM	125
Total retrieved articles		April 30, 2024	708
Number of included studies		April 30, 2024	10

### Outcome variable measures

The results from the previous studies used similar methods to measure patients' knowledge. Many articles have stated that patients exit knowledge is adequate or good if the patient answered greater than or equal to the mean score on the knowledge-related questions. And if the answer was below the mean score, then their knowledge is poor or inadequate (23, 27, 30–36). The knowledge about the drug they assessed include the drug name, the dosage, the side effects, duration, frequency, storage pace, any interaction with foods or drugs, and route of administration (23, 27, 30–36).

### Eligibility criteria

#### Inclusion and exclusion criteria

The articles included in this review evaluated the level of patient exit knowledge and associated factors with drugs dispensed in Ethiopian outpatient pharmacies. This review included studies conducted using a cross-sectional design and published in English. It also included participants from Ethiopia. It also included studies from 2014 to 2024. We excluded studies that did not address the level of patients' exit knowledge of dispensed drugs. This review excluded studies conducted outside of Ethiopia, as well as non-cross-sectional study designs.

#### Data extraction

PRISMA was used to select and direct the selection of articles for this review. Parameters used to extract data included author name, year of publication and study location, sample size for each study, study population, study design and outcome. Using a Microsoft Excel spreadsheet, we collected the necessary data from the accepted articles. Three authors (TG, TB, and CK) independently extracted information from the Supplementary Materials. Studies that met the inclusion criteria were included after deep discussion and agreement on data extraction and are summarized in the table.

#### Assessment of the risk of bias and quality

To assess the quality of the study, a critical analysis was performed using the Joanna Briggs Institute Review Meta-Analysis and Statistical Evaluation Tool. Joana identifies the studies and abstracts of the articles to decide whether they should be included. The quality of the articles was evaluated before selecting the final review. Cross-sectional studies were evaluated based on consideration of the source population, the adequacy of the sample size, data collection methods, data collection tools, statistical analysis, and adequacy of the response rate, and scored on one-to-nine-point scales. A score of a quality assessment indicator of seven or more was considered low risk for this review (Table 2).

**Table 2:** Critical appraisal results of eligible studies in this study on patients’ exit knowledge of drugs dispensed in Ethiopia, 2024 (*n* = 10).

Nigatu Hirko et al.	Q1	Q2	Q3	Q4	Q5	Q6	Q7	Q1	Q9	Total
Endalkachew M et al.	Y	Y	N	Y	Y	Y	Y	Y	Y	8
Desilu MD et al.	Y	Y	Y	N	Y	Y	Y	Y	Y	8
Ahmed teha A et al.	Y	Y	N	Y	Y	Y	Y	Y	Y	8
Tadesse Gudeta et al.	Y	Y	Y	Y	Y	Y	Y	Y	Y	9
Yabibal BT et al.	Y	N	Y	Y	Y	Y	Y	Y	Y	8
Gashaw BM et al.	Y	Y	Y	Y	Y	Y	Y	Y	Y	9
Firdawek SE et al.	Y	Y	Y	Y	Y	N	Y	Y	Y	8
Biruk W et al.	Y	Y	Y	Y	Y	N	Y	Y	Y	8
Zewdu Y et al.	Y	Y	Y	Y	Y	N	Y	Y	Y	8

Y, yes; N, no; U, unclear. JBI critical appraisal checklist for studies reporting prevalence data: Q1-was the sample frame appropriate to address the target population? Q2-Were study participants sampled appropriately? Q3-Was the sample size adequate? Q4-Were the study subjects and the setting described in detail? Q5-Was the data analysis conducted with sufficient coverage of the identified sample. Q6-Were the valid methods used for the identification of the condition? Q7-Was the condition measured in a standard, reliable way for all participants? Q8-Was there appropriate statistical analysis? Q9-Was the response rate adequate, and if not, was the low response rate managed appropriately.

#### Data processing and analysis

A Microsoft Excel spreadsheet was used to extract the data and STATA version 14 was used to analyze the data. Using random effects model analysis, the pooled level of patients' exit knowledge towards dispensed drugs in Ethiopia was calculated. With the help of a funnel chart and visual analysis, publication bias was checked. The heterogeneity of the study was tested with Cochrane Q-Static and I2. The level of exit knowledge of drugs dispensed to patients in regions was compared with an estimated prevalence using a subgroup analysis. A forest pilot with a 95% CI was used to show a pooled prevalence.

## Results

### Identification and characteristics of included studies

**Figure 1:**
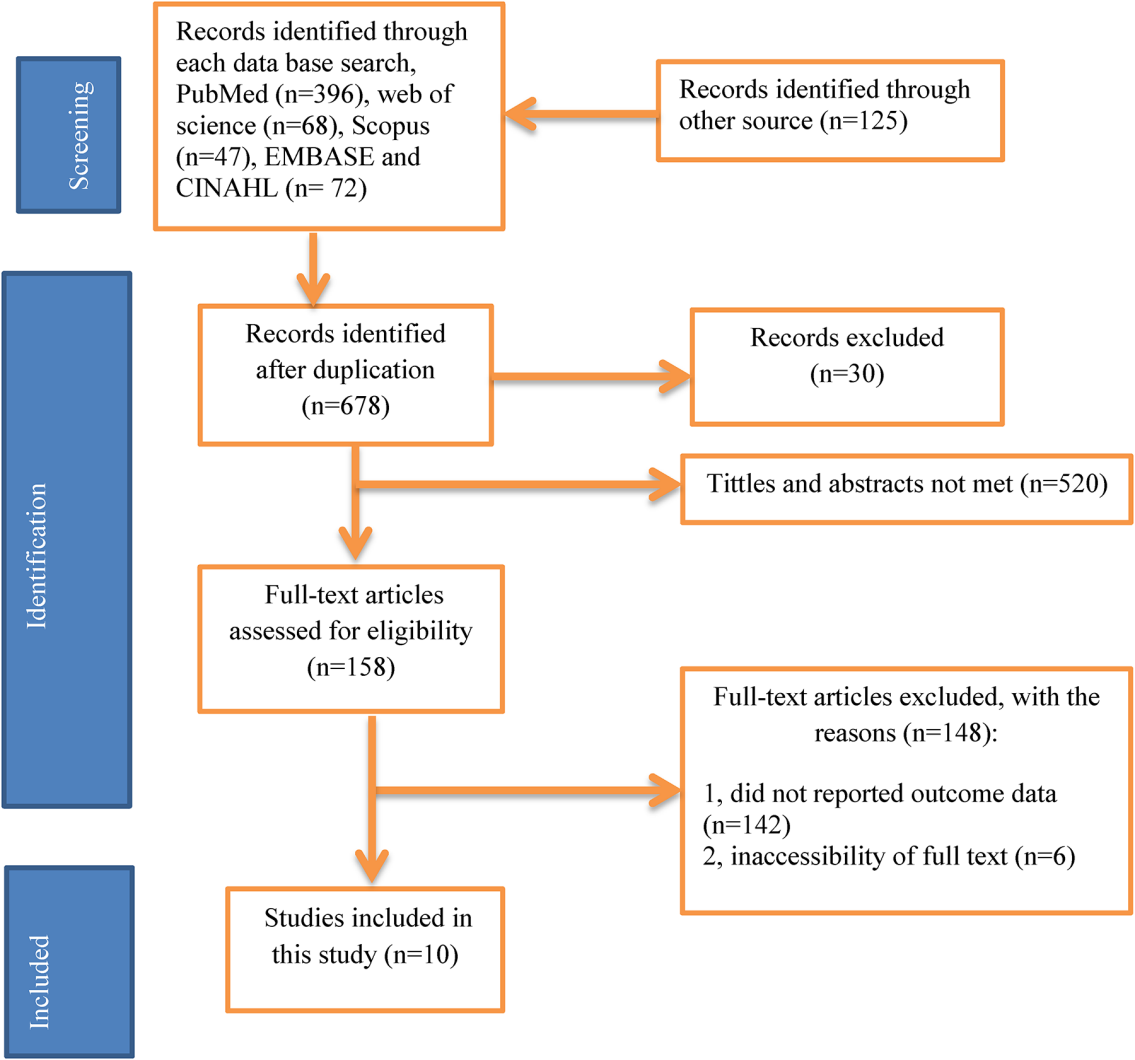
PRISMA flow diagram of study selection for systematic review of patients’ knowledge on dispensed drug in Ethiopia, 2024 (*n* = 10).

From April 1 to 30, 2024, 708 articles were identified in major electronic databases and other applicable sources. Of these identified, 30 studies were excluded due to duplicates and 678 studies were retained for further review. 520 studies were excluded because their abstracts and titles did not meet the requirements. Of the remaining 158 articles, 148 studies were excluded due to inconsistency with the inclusion criteria established for this study. Finally, 10 studies that met the eligibility criteria were included in this study (Figure 1). A total of 10 articles with 3,431 participants were included in this systematic review and meta-analysis. All included studies were cross-sectional and sample sizes ranged from 100 (31) to 422 (30). Regarding the regional distribution of the included studies, three studies were located in the Oromia region (32, 34, 35), 4 in the Amhara region (23, 31–33) in the Southern Nations, Nationalities and Peoples (SNNPR) (36), one in Tigray (27), and Harar (30) (Table 3).

**Table 3:** Study characteristics included in the systematic review of patients’ exit knowledge of drugs dispensed in Ethiopia, 2024 (*n* = 10).

Author	Year	Region	Study area	Study design	Sample	Participants	Prevalence (%)
Nigatu Hirko et al.	2019	Harar	Harar town	Cross-section	422	Patient	46
Endalkachew M et al.	2021	Oromia	Ambo	Cross-sectional	400	Patient	55.5
Desilu MD et al.	2020	Tigray	Mekele	Cross-sectional	400	Patient	81
Ahmed teha A et al.	2016	Oromia	Jimma	Cross-sectional	290	Patient	66.7
Tadesse Gudeta et al.	2019	Oromia	Jimma	Cross-sectional	357	Patient	74.8
Yabibal BT et al.	2023	Amhara	Woldia	Cross-sectional	339	Patient	46.3
Gashaw BM et al.	2020	Amhara	Gondar	Cross-sectional	402	Patient	38.3
Firdawek SE et al.	2024	Amhara	Bahirdar	Cross-sectional	318	Patient	13.3
Biruk W et al.	2020	SNNPR	Gamo	Cross-sectional	403	Patient	13.2
Zewdu Y et al.	2020	Amhara	Bahirdar	Cross-sectional	100	Patient	68

### The level of patients' exit knowledge of dispensed drugs in outpatient pharmacies in Ethiopia

**Figure 2:**
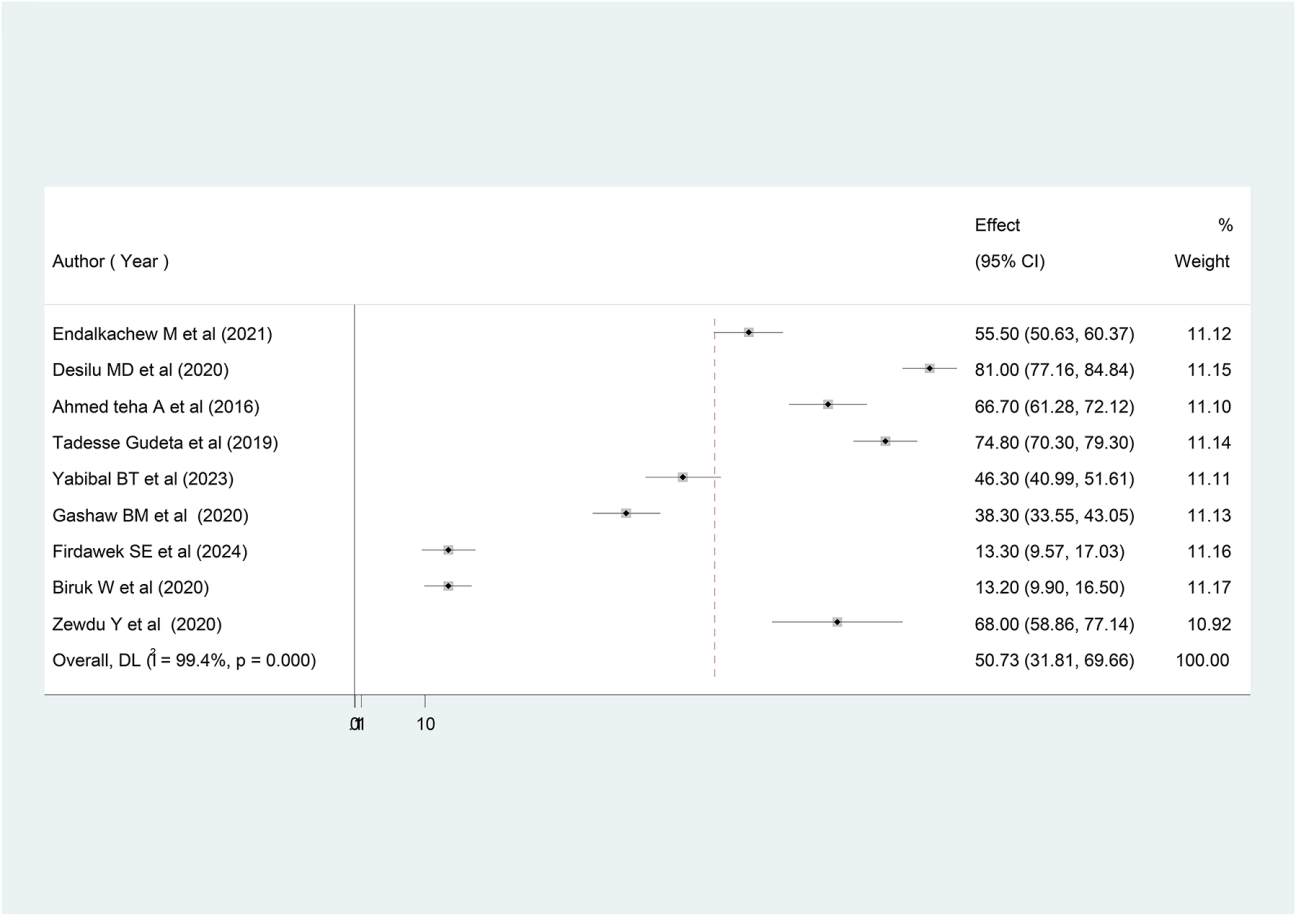
Forest plot of patients’ knowledge on dispensed drug in Ethiopia, 2024 (*n* = 10).

In Ethiopia, patients' exit knowledge of the drugs dispensed in outpatient pharmacies ranged from 13.2% (36) to 81% (27). The estimated pooled prevalence of patients' exit knowledge of dispensed drugs was 50.73% [95% CI (31.81; 69.66); I2 = 99.4%, *P* < 0.001] (Figure 2).

### Subgroup analysis of exit knowledge of patients about drugs dispensed in outpatient pharmacies in Ethiopia

**Figure 3:**
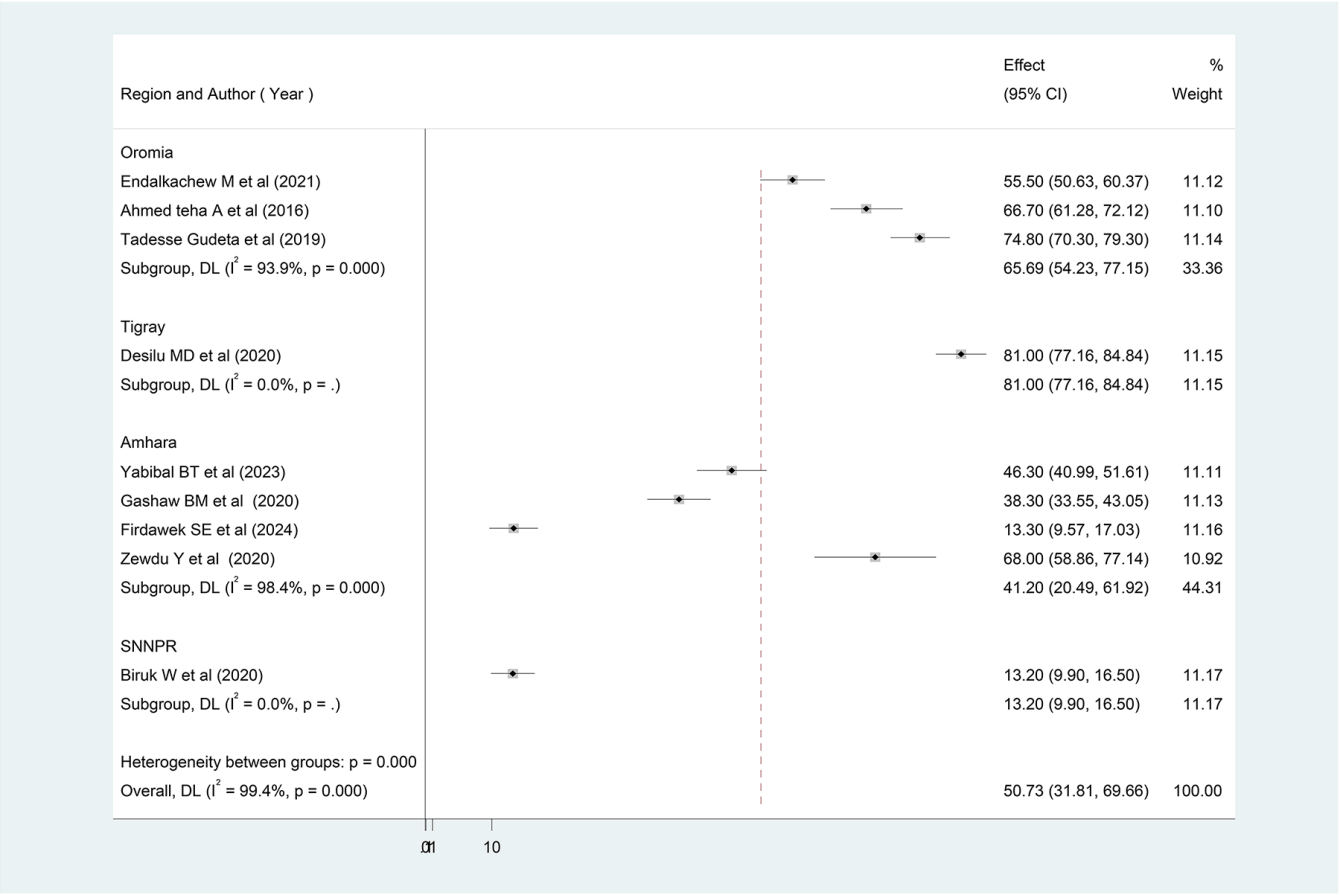
Subgroup analysis of patients’ knowledge on dispensed drug in Ethiopia, 2024 (*n* = 10).

Tigray had the highest level of patients’ exit knowledge of drugs dispensed with a score of 81.0% (95% CI 77.16, 84.84), followed by Oromia with 65.69% (95% CI 54.23, 77.15), and the lowest value was observed in the SNNPR at 13.20% (95% CI 9.90, 16.50) (Figure 3).

### Heterogeneity and publication bias

**Figure 4:**
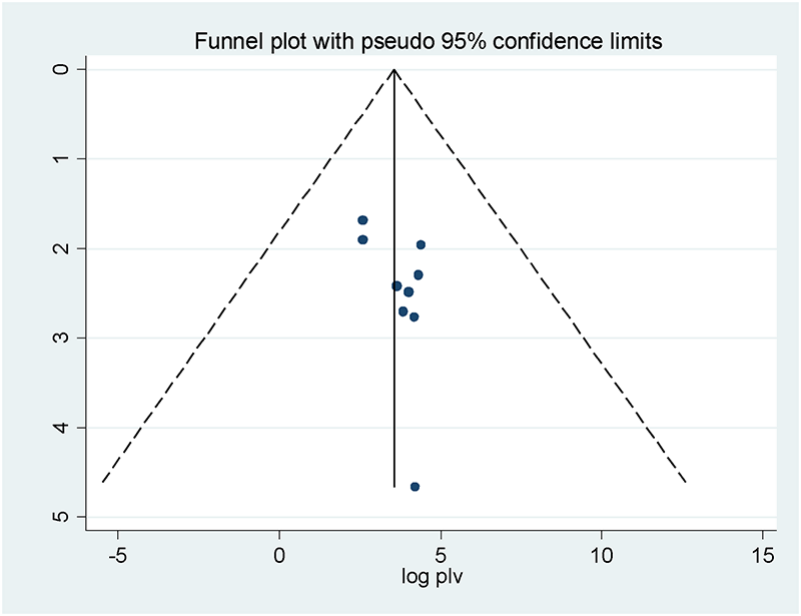
Funnel plot of patients’ knowledge on dispensed drug in Ethiopia, 2024 (*n* = 10).

To reduce and balance the study's heterogeneity, we performed a subgroup analysis by region. The results of the I2 test show significant heterogeneity between studies (I2 = 99.4%, *P* < 0.001). Eggers' test and visual inspection of funnel plot were used to confirm the study publication. The funnel plot revealed that the selected studies had a symmetrical distribution after inspection (Figure 4) and Eggers' test (*P* = 0.108). This demonstrated that there was no sign of publication bias.

### Factors associated with patients' exit knowledge of drugs dispensed in Ethiopia

In this review, two variables (patient received adequate information from the dispenser [OR = 12.21, CI (1.38, 136.8), I2 = 88.2%, *p* = 0.004] and level of education [OR = 10.32, CI (1.33, 79.9), I2 = 82.5%, *p* = 0.001] were significantly associated with patient exit knowledge of the drugs dispensed, while occupation status [*P* = 0.215] and severity of the health condition [*P* = 0.490] were not significantly associated with patient exit knowledge.

The review revealed a significant association between the level of education of patients and their knowledge of the drugs dispensed. Higher levels of education of patients were 10.32 times more likely to have a good exit knowledge of the patient than the contrast group [OR = 10.32, CI (1.33, 79.9), I2 = 82.5%, *p* = 0.001]. This review also found a significant association between women who received adequate information on dispensed drugs and the exit knowledge of patients. Patients' exit knowledge of drugs dispensed was almost 12 times higher among patients who received adequate information on the drugs dispensed than in those group who did not received adequate information[OR = 12.21, CI (1.38, 136.8), I2 = 88.2%, *p* = 0.004] (Table 4).

**Table 4:** Factors associated with prevalence of patients’ exit knowledge of drugs dispensed of the systematic review and meta-analysis in Ethiopia (*n* = 10).

Factors	OR	CI	I^2^	*P*-value	Significance level
Received adequate information (yes)	12.21	[1.38, 136.8]	81.2%	0.001	Significance
Educational level (secondary and above)	10.32	[1.33, 79.9]	82.5%	0.001	Significance
Occupation (unemployed)	0.29	[−0.07, 0.66]	34.9%	0.215	Non-significance
Severity of health condition (yes)	4.43	[1.6, 7.26]	87.4%	0.490	Non-significance

### Review outcomes

The first outcome of this systematic review and meta-analysis was an estimate of the overall level of knowledge of dispensed medications in Ethiopia at hospital discharge. The second outcome concerns factors related to patients' knowledge of the medications dispensed at discharge.

## Discussion

In this systematic review and meta-analysis, the pooled level of patient exit knowledge of drugs dispensed in Ethiopia was 50.73% [95% CI (31.81; 69.66); I2 = 99.4%, *P* < 0.001]. This study is supported by a previous study (23). However, this result is higher than previous studies conducted in Sri Lanka (17.5%) (28), Ghana (31%) (29), Gondar city (38.3%) (23), Gamo (13.2%) (35) and lower than studies conducted in Mekele (81%) (27), 66.7% in Jimma (35), 68% in Amhara (31), and 55.5% in Ambo (32). Differences could be explained by study participants' socio-demographic characteristics, policies and strategies, women's perceptions, health facility drug supply, study period, location, methodology, quality of pharmacy services, study approach, healthcare access, and patients' attitudes and knowledge.

The Tigray region had the highest patient exit knowledge, while the SNNPR region had the lowest values. This could be due to differences in pharmaceutical care standards, compassionate and respectful patient care practices, and dispenser attitudes and expectations. Additionally, disparity in patients' knowledge level of the included study and the healthcare providers' experience in dispensing could be the reasons for the difference.

Patients' educational status and adequate information provided to patients were identified as significant factors associated with the patients' exit knowledge. Educational level of the respondents was significantly associated with their knowledge of the drugs dispensed. Respondents with higher levels of education were more likely to be knowledgeable about the drugs dispensed than respondents who could not read or write. This result is similar to previous research (23, 28, 37). This suggests that higher levels of education can facilitate communication between patients and dispensers, which can influence their perceptions of access to and use of dispensed medicines. This implies that the Ministry of Education and other stakeholders must encourage community access to higher education. Therefore, the patient's level of education needs special attention during the consultation and provision of pharmaceutical services (37). For illiterate patient, the dispenser should provide adequate information by repeating the required information or word many times until they confirm that the patients understand what they told to them. In addition, the dispenser should write the most important information such as dose, frequency, and route on the drug strip or paper that the patient can ask other educated family members.

The probability of knowing what drug was dispensed was highest among patients who received complete information from the pharmacist. The results of this study are consistent with studies conducted in Botswana (38) and Ethiopia (30, 37, 38). This may be explained by the greater emphasis on pharmacist training in patient-centered pharmacy services (39). This implies that stakeholders must provide on-the-job training to pharmacy technicians in patient-centered pharmacy services (40). In addition, several studies demonstrate the importance of pharmacist participation in ongoing patient counseling to significantly improve patient knowledge and their compliance with drug (40–43). Therefore, this implies that pharmacists who interact with their patients through clear instructions can positively influence patient attitudes and patient awareness of the drug (s) dispensed.

This review has certain limitations: It included only cross-sectional studies that did not assess cause-and-effect analysis; the primary studies in this review did not include all 12 regions, and the majority of the included studies relate to specific regions of the country, which can make it difficult to generalize results for all regions in Ethiopia. Additionally, while subgroup analysis can helped in identifying potential causes of heterogeneity (e.g., regional differences in healthcare quality, patient demographics, or pharmacy practices), it does not necessarily “balance” or reduce heterogeneity. While significant publication bias appears unlikely, other biases such as selection bias (e.g., exclusion of non-English studies) or methodological inconsistencies across studies, could still affect the results.

## Conclusion

1 in 2 patients (50%) had good knowledge about the drugs dispensed in Ethiopia. Adequate drug information provided to patients and their educational level were important predictors of patients’ exit knowledge about dispensed medications. Poor knowledge of the patient's exit will affect his recovery. Therefore, training of dispensers, providing adequate advice on the dispensed medicine, improving patient knowledge about the medicine, how and when to use the medicine are strongly recommended for the dispenser, Ministry of Health, and other nongovernmental organizations.

## Data Availability

The original contributions presented in the study are included in the article/Supplementary Material, further inquiries can be directed to the corresponding author.
